# What’s the remedy for the distal necrosis of DIEP flap, better venous drain or more arterial supply?

**DOI:** 10.1371/journal.pone.0171651

**Published:** 2017-02-10

**Authors:** Yi Zhang, Tingliang Wang, Jiao Wei, Jinguang He, Tao Wang, Ying Liu, Hua Xu, Jiasheng Dong

**Affiliations:** Shanghai Ninth People's Hospital affiliated to Shanghai Jiao Tong University School of Medicine, Shanghai, China; di Pompeo d'Illasi, Universita degli Studi di Roma La Sapienza Facolta di Medicina e Psicologia, ITALY

## Abstract

**Background:**

We developed a novel pedicled DIEP flap model in rat to explore the possible remedy for the distal necrosis of the flap.

**Methods:**

A deep inferior epigastric perforator (DIEP) flap, based on the second right cranial perforator (P2) as the main pedicle, was elevated in 48 Sprague-Dawley rats. The rats were randomized into 4 groups: group I, the left P2 remaining intact as supercharging; group II, the left P2 artery alone kept as supercharging; group III, the left P2 vein alone kept as supercharging; group IV, no supercharging. Transcutaneous oxygen pressure (TcPO_2_) and transcutaneous carbon dioxide pressure (TcPCO_2_) were measured immediately after flap elevation, protein level of Hif-1a was measured 48 hours later, and flap survival was assessed 7 days postoperatively.

**Results:**

Blockade of artery led to significantly lower TcPO_2_, higher TcPCO_2_, and higher expression level of Hif-1a in the distal side of the flap in group III and group IV, than those of group I and group II. At 7 days post surgery, significantly lower flap survival rates were observed in group III (81.9 ± 5.7%) and group IV (78.4 ± 6.5%), compared to observed in group I (97.2 ± 3.0%) and group II (94.2 ± 6.2%).

**Conclusions:**

It might be arterial insufficiency, not venous congestion, which mainly caused the distal necrosis of the DIEP flap in rat. Arterial instead of venous supercharging might be a more effective procedure that improves circulation to zone IV of the flap.

## Introduction

The deep inferior epigastric perforator (DIEP) flap has become an increasingly popular flap choice for the reconstructive surgeries [1–3], since its first introduction in 1989 by Koshima et al [4]. However, one of the main drawbacks of the traditional DIEP flap is the compromised circulation in its distal segment area (the classic Hartrampf zone IV), which might lead to ischemia and tissue loss [5,6]. Discarding zone IV is a routinely applied procedure, but it may limit transferrable tissue.

Vessel supercharging is a useful technique providing a solution to distal necrosis in a free flap transplantation [7]. Our previous work has demonstrated the reconstruction of a ptotic breast using double-pedicle DIEP flap, in which a contralateral perforator (including an artery and venae comitante) is preserved as supercharging by microvascular anastomosis [8,9]. However, the relative importance of arterial versus venous supercharging in enhancing survival of the flap remains controversial. Some literatures presumed that the venous congestion is the main cause of the zone IV necrosis of DIEP or TRAM flaps [10,11], while other literatures demonstrated that the arterial inflow had a marked effect on the survival of the distal part of the flaps [12,13]. In order to explore the physiology and hemodynamics of the DIEP flap, a lot of experimental animal models have been developed, but the conclusions were still conflictive [7,14–17].

In the present study, we developed a novel DIEP flap model in rat, in which a contralateral perforator is preserved for augmenting arterial supply or venous drainage. We aimed to explore the potential remedy for distal part necrosis of the rat perforator flap, by observing and comparing the efficacy of different distal vessel supercharging.

## Materials and methods

All experimental and animal care procedures were in compliance with NIH Guiding Principles for Research Involving Animals and were approved by the Institutional Animal Care and Use Committee of Shanghai Jiao Tong University School of Medicine (protocol number: HKDL[2016]41). Forty eight male Sprague-Dawley (SD) rats, 10–12 week old, weighing between 280g to 300g, were randomized into four groups (n = 12 each): group I: arterial and venous supercharged; group II: arterial supercharged; group III: venous supercharged; group IV: no supercharging, served as the control. Postoperatively, rats were housed individually and fed standard rat chow and water adlibitum upon completion of the experiment. The food and water were placed inside the cage so that the animals did not have to stand on their hind legs to reach them. All the animals were sacrificed by a lethal dose (100 mg/kg) of intracardiac Nembutal, after the observations were completed.

### Surgical procedure

**Fig 1:**
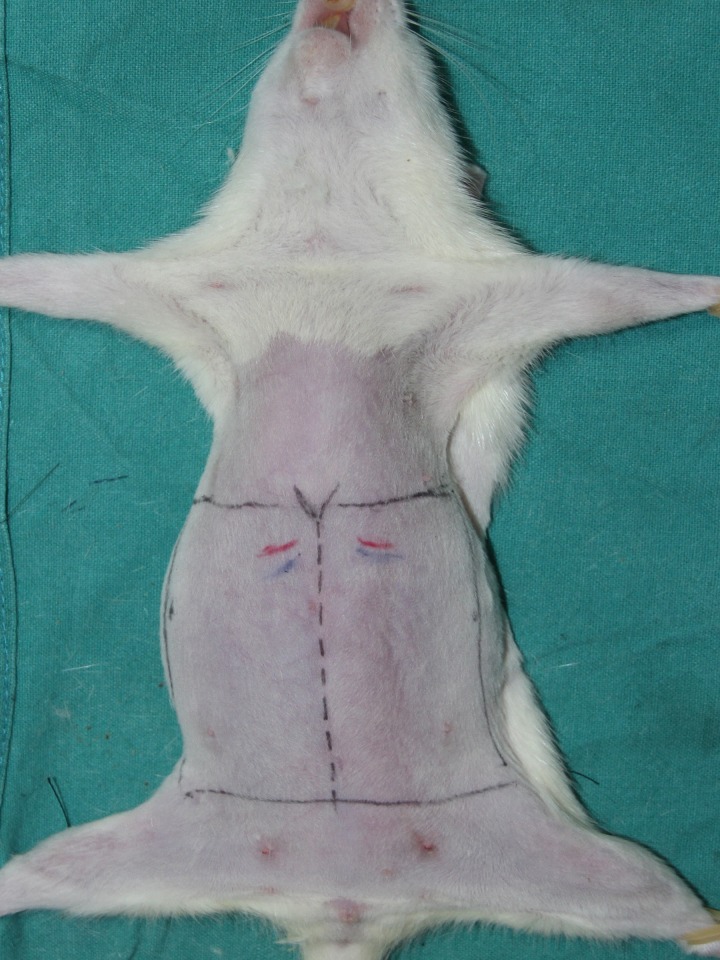
Design of the flap model in rat. The superior margin was horizontal to the tip of the xiphoid. The inferior border was parallel to this and joined the anterosuperior iliac spine just above the pubis. The rectangular shape of the flap was effected with two verticallines at the posterior axillary folds.

Hair on the abdomen was removed with an electric razor after rats were anesthetized with pentobarbital sodium (50 mg/kg, intraperitoneal). The abdominal flap model described by Oksar et al was used to fabricate a DIEP flap [18]. The flap was designed according to the anatomic landmarks, and the dimensions of the flaps were between 3.6 × 7.5 cm and 3.8 × 8.5 cm (range, 27.0 to 32.3 cm^2^). The superior margin was horizontal to the tip of the xiphoid. The inferior border was parallel to this and joined the anterosuperior iliac spine just above the pubis. The rectangular shape of the flap was effected with two vertical lines at the posterior axillary folds (Fig 1).

**Fig 2:**
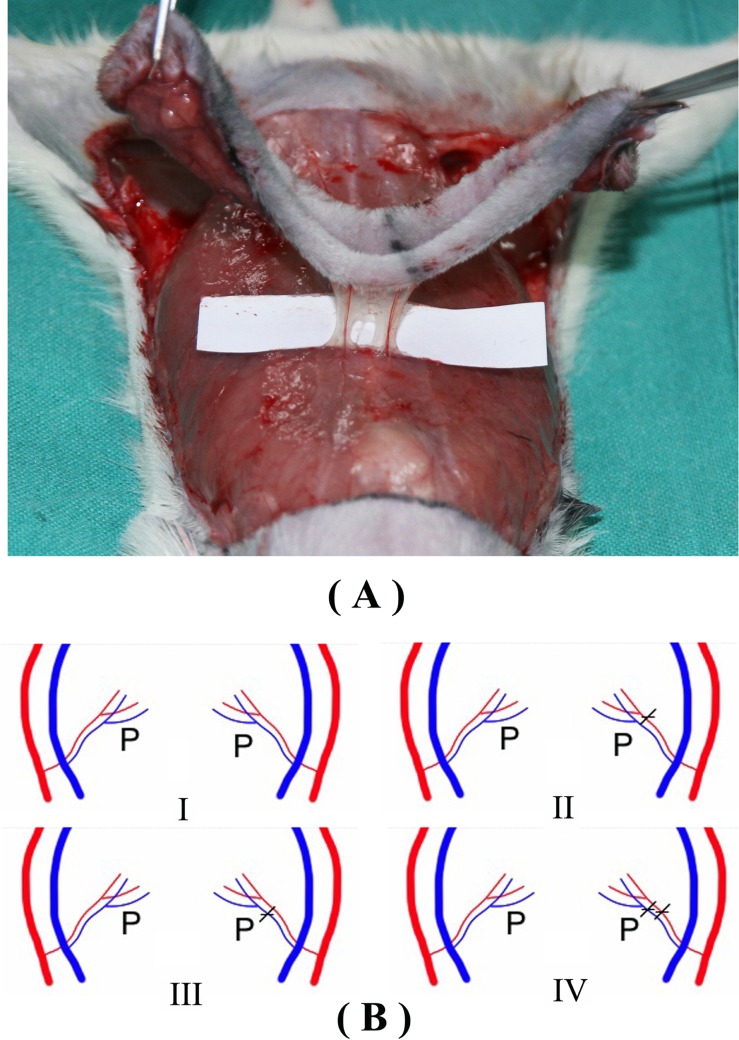
Schematic of the supercharging for the DIEP flaps in each group. **(A)** The second cranial perforator (P2) from both the right and left rectus abdominis muscle are found and carefully isolated. **(B)** Rats are randomized into 4 groups: Group I, the left P2 remaining intact as supercharging; Group II, the left P2 artery alone kept as supercharging; Group III, the left P2 vein alone kept as supercharging; Group IV, no supercharging.

**Fig 3:**
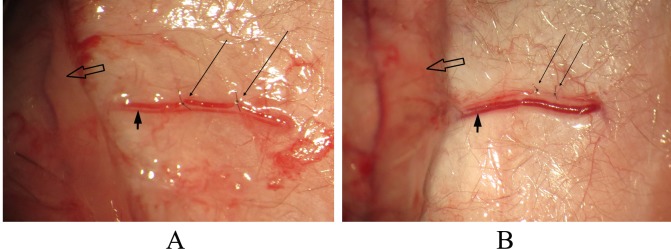
Arterial/venous ligation under the microscope. **(A)** In group II, the vein of the left P2 (coarse arrow) is found microscopically, and ligated twice by 11–0 suture (long slender arrow). **(B)** In group III, the artery of the left P2 (coarse arrow) is found microscopically, and ligated twice by 11–0 suture (long slender arrow); Hollow arrow indicates the DIEP flap.

The second cranial perforators (P2) from the right and left rectus abdominis muscle were identified and carefully isolated (Fig 2A). The right P2 was protected as the main pedicle in all flaps (Fig 2B). In group I, the left P2 were remained intact as supercharging. In group II, the vein of the left P2 was ligated twice by 11–0 suture under an operating microscope, with the perforator artery left as an arterial supercharging (Fig 3A). In group III, the artery of the left P2 was ligated twice by 11–0 suture, with the perforator vein left as a venous supercharging (Fig 3B). In group IV, all the cranial perforators from the left rectus abdominis muscle were cut and coagulated with a bipolar. All the flaps were sewn back into place, after the vascular patency of the pedicles was confirmed microscopically.

### TcPO_2_ and TcPCO_2_

**Fig 4:**
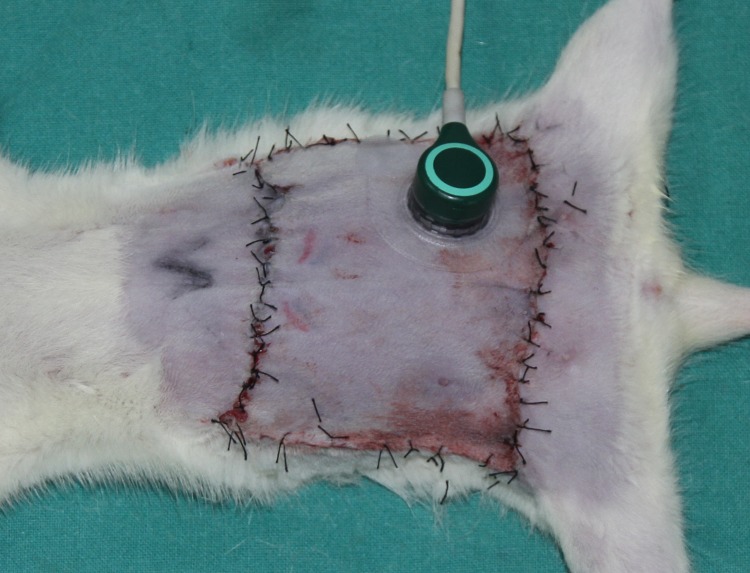
Measurement of TcPO_2_/TcPCO_2_. The transcutaneous sensors is calibrated and attached to the surface of flap skin.

Transcutaneous oxygen pressure (TcPO_2_) and transcutaneous carbon dioxide pressure (TcPCO_2_) were measured before and 20 minutes after flap elevation, on both the proximal and distal sides of the flap, using a TCM4 monitor (Radiometer Ltd, Copenhagen, Denmark). The transcutaneous sensors were calibrated and placed on the flap skin per the manufacturer’s recommendations V (Fig 4) and the data was collected at same sites for each flap.

### Western blot analysis

All biopsy specimens were obtained from that part of the skin flap, and each biopsy specimen was harvested with a small pair of microsurgical scissors with a sample size of 1cm^3^. Protein was extracted from specimens with RIPA buffer containing protease inhibitors, separated by SDS-polyacrylamide gel electrophoresis and transferred onto nitrocellulose membranes. The membranes were incubated overnight at 4°C with anti-Hif-1a Ab (1:1000 dilution; sc-13515, Santa Cruz Biotechnology, USA) and anti-β-actin Ab (1:1,000 dilution; CP01; Calbiochem, San Diego, CA, USA), followed by incubation with corresponding HRP-conjugated secondary antibodies. Signals were detected in a sensitive digital imaging equipment (Image Quant LAS 4000 mini; GE Healthcare Bio-Sciences AB, Uppsala, Sweden) using a commercial ECL detection kit (Millipore, Billerica, MA).

### Immunohistochemistry

Bbiopsy specimens were fixed in 4% paraformaldehyde, embedded in paraffin, and then sliced as 4 μm sections. Immunochemistry was performed using an antibody against Hif-1a (1:400 dilution; sc-13515, Santa Cruz Biotechnology, USA) with overnight incubation at 4°C, followed by incubation with HRP-conjugated secondary antibodies (1:400 dilution; KIT-5108; Maixin, Fujian, China). Sections were imaged using a Zeiss AxioImager Standard Microscope (Carl Zeiss, Berlin, Germany).

### Flap survival analysis

At the 7^th^ day after flap elevation, the animal was reanesthetized and the appearance of the skin flap was recorded and photographed in a standardized fashion. The flap viable area was determined based on color, capillary refill, and the pin-prick test. Ratios of viable area to original flap area (measured immediately after sewn) were calculated by digital planimetry software (Image-Pro Plus Version 7.0), expressed as a percentage (percent survival).

### Statistical analysis

Data are presented as mean ± standard deviation (SD). The two-tailed t-test was employed to draw a comparison between groups. Differences are considered as significant when the P-value is under 0.05.

## Results

**Fig 5:**
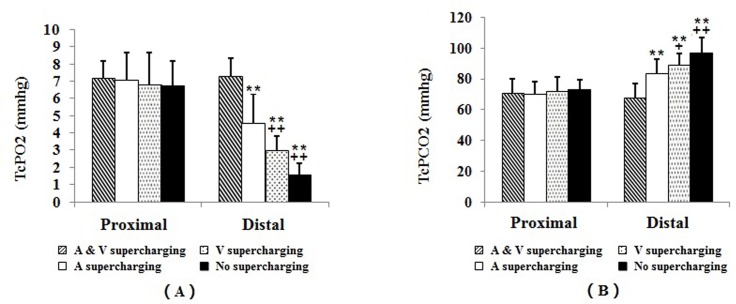
TcPO_2_ and TcPCO_2_ values in the flaps 20 minutes post elevation. In group II, III and IV, significantly lower TcPO_2_ with higher TcPCO_2_ are observed on the distal side of the flap, than observed on the proximal side. Compared to group I, group II, III, and IV shows significantly lower TcPO_2_ with higher TcPCO_2_ on the distal side of flaps. Compared to group II, group III and IV shows further lower TcPO_2_ with further higher TcPCO_2_ on the distal side of flaps; **: P < 0.01 vs. group I; ^**++**^: P < 0.01 vs. group II.

The baseline levels of TcPO_2_ and TcPCO_2_ on the abdominal skin surface showed no significant difference among the four groups. At 20 minutes after flap elevation, significant decrease in TcPO_2_ and significant increase in TcPCO_2_ were observed on both sides of the flaps in all the four groups. In group II, III and IV, significantly lower TcPO_2_ with higher TcPCO_2_ were observed post elevation on the distal side of the flap, than observed on the proximal side. Compared to supercharging with intact P2 (group I), blockage of either P2 vein (group II) or artery (group III) led to significantly lower TcPO_2_ with higher TcPCO_2_ on the distal side of flaps. Compared to arterial supercharging with (group II), venous supercharging (group III) and no supercharging (group IV) led to further lower TcPO_2_ with further higher TcPCO_2_ on the distal side of flaps (Fig 5).

**Fig 6:**
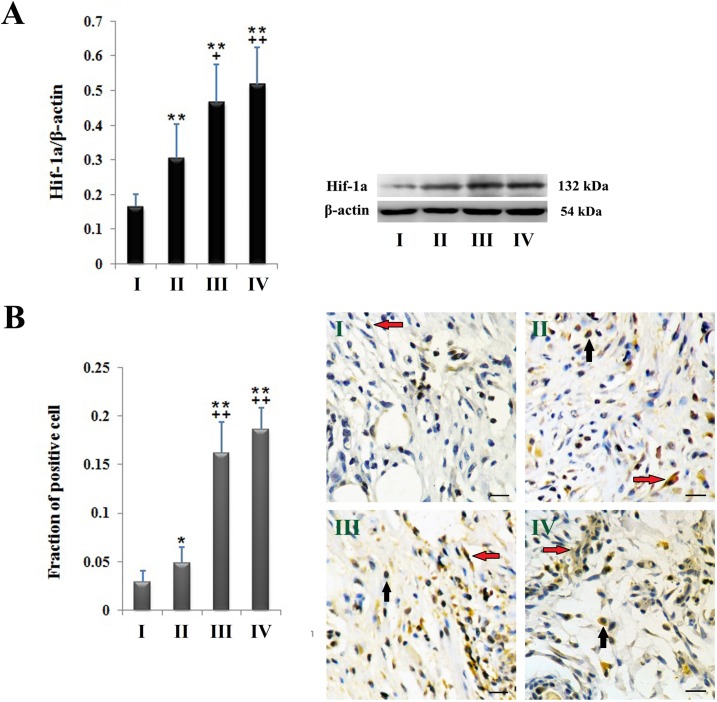
Hif-1a expression in the distal side of flaps 48 hours post elevation. **(A)** Western blot shows significantly higher level of Hif-1a expression in zone IV of the flap in group II, III and IV than that of group I. **(B)** Immunohistochemistry shows strongly positive staining for Hif-1a in the dermis of flap tissues of the groups without arterial supercharging (group III and IV), compared to that of P2 arterial supercharged groups (group I and II); Red arrow: fibroblasts of positive Hif-1a staining; Black arrow: macrophages/mononuclear neutrophils of positive Hif-1a staining; Bars = 20mm; *: P < 0.05 vs. group I; **: P < 0.01 vs. group I; ^**+**^: P < 0.05 vs. group II; ^**++**^: P < 0.01 vs. group II.

Hif-1a expression was induced in flap tissues of all groups 48 hours post elevation. Compared to group I, group II, III and IV showed significantly higher expression level of Hif-1a in the distal side of the flap. Further higher expression of Hif-1a was observed in group III and IV than that of Group II (Fig 6A). The results were confirmed by immunohistochemistry. As shown in Fig 6B strongly positive staining for Hif-1a was seen in the dermis of flap tissues of the groups without arterial supercharging (group III and IV), compared to that of arterial supercharged groups (group I and II).

Flaps from all groups were flattened to measure the percentage of survival area relative to the flap total area 7 days post elevation, as listed in Table 1. Compared to group I (97.2 ± 3.0%) and group II (94.2 ± 6.2%), significantly lower flap survival rates were observed in group III (81.9 ± 5.7%) and group IV (78.4 ± 6.5%). In contrast, difference in flap survival rate between group I and group II, and between group III and group IV were not significant. In groups without arterial supercharging (group III and IV), a pattern of necrosis contralateral to the main pedicle (zone IV) was observed as described by others authors.

**Table 1:** Flap survival rates in each group at the 7th day.

Groups	*A&V Sup*	*A Sup*	*V Sup*	*No Sup*
**Survival Rate** *Avr*. *± SD (%)*	*97*.*2 ± 3*.*0*	*94*.*2 ± 6*.*2*	*81*.*9 ± 5*.*7*	*78*.*4 ± 6*.*5*
***P***_***1***_	*-*	*0*.*15*	***< 0*.*01***	***< 0*.*01***
***P***_***2***_	*-*	*-*	***< 0*.*01***	***< 0*.*01***
***P***_***3***_	*-*	*-*	*-*	*0*.*18*

A: Arterial; V: Venous; Sup: Supercharging; P_1_: vs. A&V Sup; P_2_: vs. A Sup; P_3_: vs. V Sup; p value in bold when less than 0.05.

## Discussion

The outcomes of the present study indicate that an intact contralateral perforator can provide sufficient supercharging to the zone IV of a rat DIEP flap. Ligation of the perforator venae comitante does not significantly weaken the supercharging effect, while ligation of the perforator artery will significantly endanger the survival of the distal flap.

With more advantages over other traditional flaps, DIEP flap has been preferred as a “gold standard” for autologous microsurgical breast reconstruction. In case of patient with media/large breast and inadequate zone I-III flap volume, contralateral mastopexy or reduction are routine procedures performed in Western patients. However, Oriental females usually have thinner abdominal wall, and are often reluctant to have their unaffected breast surgically manipulated due to cultural considerations. For them, several second-tier options to augment a traditional DIEP flap could be considered. DIEP flap in combination with prosthetic implants can achieve an adequate volume with minimally increased operative time, but is less desirable for patient who wishes to avoid the use of implants [19]. The adjunct of fat grafting as a secondary procedure expands the indications of DIEP flap reconstruction in patients with insufficient donor-site volume, but commits the patients to separate operations spaced several months apart [20]. Agarwal and Gottlieb reported on a series of 14 patients where bipedicled flaps using combinations of DIEP and transverse rectus abdominis myocutaneous (TRAM) dissections allowed for safe transfer of zones I-IV in women with large breast, thin abdominal tissue, or with midline scars [21]. Stacked DIEP flap reconstruction is another option, a safe and innovative technique to achieve an adequate volume and projection, but its major drawback is the increased complexity of the anastomoses [22].

Supercharging anastomoses of arteries/veins is a useful technique that increases flap vascularization. Arterial supercharging with additional arteries increases flap perfusion, while venous supercharging with additional veins increases flap drainage. With additional anastomosis, shortage of blood supply in the distal area can get great relieve [8,23–26]. However, the relative importance of arterial versus venous supercharging in enhancing DIEP flap survival is of wide debates and the conclusions of literatures are conflicted on the animal experiments. Hallock et al. [14] and Groth et al. [15] proposed that venous supercharging of a rat DIEP flap ensured greater flap survival. But in their experiments only venous supercharging was included, while arterial supercharging had not been tested. Using a rat DIEP model supercharged by superficial inferior epigastric (SIE) vessels, Gumus et al. stated either venous or arterial supercharging could enlarge the survival area of the flap [17]. Based on the same animal model, the finding of Yamamoto indicated that the major factor contributing to distal necrosis of the DIEP flaps was arterial insufficiency rather than venous congestion [16].

Two issues need to be solved to get the experiments more plausible. First, in most of the previous literatures, SIE vessels were chosen as the supercharging of the DIEP models. Human being known as fixed skinned animal possess abdominal skin and subcutaneous tissues which are supplied primarily by perforators coursing through the underlying muscles [27,28]. Compared with loose skinned animal including rats, the SIE vessels of human are relatively small-caliber and not constant in clinical setting [29,30]. Thus, success or failure based on those models might not imply that supercharging of SIE vessels will be practicable in human being. Thus, in the present study, a contralateral perforator was selected for supercharging, so as to simulate the bipedicle DIEP flap performed in our clinical routine [31]. The outcomes of the flap model supercharged with an intact perforator (survival rate: 97.2%) and the flap model without supercharging (survival rate: 77.2%) are consistent with our clinical experience with transfer of double-pedicle and single-pedicle DIEP flaps, which indicates the predictive value of the model.

Another issue to solve is selection of flap monitoring strategies. In the previous studies, monitoring methods including temperature monitoring, measurements of vascular pressure, and scanning laser Doppler imaging have been investigated as indices of perfusion to predict flap survival [16,17,32]. However, in some cases, it is hard to tell whether flaps are oxygenized or only perfused. TcPO_2_ is a non-invasive monitor which is sensitive to changes of tissue oxygen [32]. In the present study, our outcomes indicates that arterial supercharging benefits zone IV of the DIEP flap with higher level of TcPO_2_ and lower level of TcPCO_2_ than venous supercharging does in the early phase after flap elevation. Hif-1 is a heterodimeric transcription factor, composed of α and β subunits. Hif-1α expression increase exponentially when mammalian cells are subjected to hypoxia [33]. Consistent with TcPO_2_ monitoring results, lower expression level of Hif-1a was observed 48 hours after flap elevation at zone IV when arterial rather than venous supercharging was used, implying its higher anti-ischemia efficacy.

As we know, arterial perfusion and venous drainage are the two successive steps which may affect tissue oxygen and nutrition supply, and in turn affect the survival of the flaps. The preliminary evidence from this present study implies that the major factor contributing to zone IV ischemia in this experimental DIEP model is the arterial inflow rather than the venous outflow, and arterial supercharging significantly decrease the distal flap necrosis, while venous supercharging alone showed no significance to improve the flap viability. Our result related to arterial verse venous supercharging was similar to the previous experimental studies. However, it doesn’t mean venous congestion could be perceived less as a threat to the flap, since perfusion related complications might be multi-factorial in clinical practice. In a recent retrospective study, Mohan et al. [34] presented an algorithm to maximize efficiency and safety for perforator selection in DIEP flap reconstruction. Factors listed in relation to intra-operative decision-making included size and weight of the flap, number and type of the perforators, body mass index of patients, and the skill and clinical experience of surgeons. Rubino et al. [35] demonstrated that the bigger is the flap the greater is its flow rate, and consequently its venous drainage needs to be higher. In rare cases of our experience, patients grafted with DIEP flap may develop acute venous congestion even supercharging with contralateral DIEV is performed. This might be result from lack of anastomoses between the superficial and deep venous system. In such situation, blood that fills capillary cannot get into deep venous system, which leads to venous ischemia, and contralateral SIEV supercharging is recommended as a salvage procedure.

## Supporting information

S1 Table TcPO_2_ and TcPCO_2_ values on the proximal side of flaps. (DOCX)

S2 Table TcPO_2_ and TcPCO_2_ values on the distal side of flaps. (DOCX)

S3 Table Fraction of Hif-1a positive cell in the distal side of flaps (Immunohistochemistry). (DOCX)

S4 Table Hif-1a expression in the distal side of flaps (Western blot). (DOCX)
