# Supplementary material for: What’s the remedy for the distal necrosis of DIEP flap, better venous drain or more arterial supply?
Source: PLoS One. 2017 Feb 10;12(2):e0171651. doi: 10.1371/journal.pone.0171651 (PMC5302794; doi:10.1371/journal.pone.0171651)
Supplement: S1 Table — (DOCX) [file pone.0171651.s001.docx]

**S1 Table. TcPO_2_ and TcPCO_2_ values on the proximal side of flaps.**

|  | **TCPCO_2_** | | | | **TCPO_2_** | | | |
| --- | --- | --- | --- | --- | --- | --- | --- | --- |
|  | Group I | Group II | Group III | Group IV | Group I | Group II | Group III | Group IV |
| **Mean** | 7.183 | 7.050 | 6.817 | 6.733 | 70.817 | 69.600 | 72.333 | 73.300 |
| **SD** | 1.844 | 1.569 | 1.808 | 1.449 | 9.519 | 8.214 | 7.952 | 6.421 |
