# Supplementary material for: What’s the remedy for the distal necrosis of DIEP flap, better venous drain or more arterial supply?
Source: PLoS One. 2017 Feb 10;12(2):e0171651. doi: 10.1371/journal.pone.0171651 (PMC5302794; doi:10.1371/journal.pone.0171651)
Supplement: S2 Table — (DOCX) [file pone.0171651.s002.docx]

**S2 Table. TcPO_2_ and TcPCO_2_ values on the distal side of flaps. .**

|  | **TCPO_2_** | | | | **TCPCO_2_** | | | |
| --- | --- | --- | --- | --- | --- | --- | --- | --- |
|  | Group I | Group II | Group III | Group IV | Group I | Group II | Group III | Group IV |
| **Mean** | 7.333 | 4.567 | 3.017 | 1.567 | 68.417 | 83.367 | 89.567 | 97.050 |
| **SD** | 1.227 | 1.726 | 0.821 | 0.628 | 9.160 | 9.255 | 6.945 | 10.043 |
