# Supplementary material for: What’s the remedy for the distal necrosis of DIEP flap, better venous drain or more arterial supply?
Source: PLoS One. 2017 Feb 10;12(2):e0171651. doi: 10.1371/journal.pone.0171651 (PMC5302794; doi:10.1371/journal.pone.0171651)
Supplement: S3 Table — (DOCX) [file pone.0171651.s003.docx]

**S3 Table. Fraction of Hif-1a positive cell in the distal side of flaps (Immunohistochemistry).**

|  | Group I | Group II | Group III | Group IV |
| --- | --- | --- | --- | --- |
| **Mean** | 2.88% | 4.87% | 16.13% | 18.63% |
| **SD** | 1.14% | 1.61% | 3.27% | 2.19% |
