# Supplementary material for: What’s the remedy for the distal necrosis of DIEP flap, better venous drain or more arterial supply?
Source: PLoS One. 2017 Feb 10;12(2):e0171651. doi: 10.1371/journal.pone.0171651 (PMC5302794; doi:10.1371/journal.pone.0171651)
Supplement: S4 Table — (DOCX) [file pone.0171651.s004.docx]

**S4 Table. Hif-1a expression in the distal side of flaps (Western blot).**

|  | Group I | Group II | Group III | | Group IV |
| --- | --- | --- | --- | --- | --- |
| **Mean** | 0.165 | 0.305 | 0.465 | 0.517 | |
| **SD** | 0.036 | 0.099 | 0.111 | 0.108 | |
